# Effect biomonitoring in a controlled firefighting setting: an untargeted metabolomic pilot study

**DOI:** 10.1007/s00204-026-04397-w

**Published:** 2026-04-25

**Authors:** Max-Philipp Boehler, Christian Kersch, Bernd Rossbach, Andrea Kaifie, Simone Schmitz-Spanke

**Affiliations:** 1 Institute and Outpatient Clinic of Occupational, Social, and Environmental Medicine, Friedrich-Alexander-University of Erlangen-Nuremberg, Henkestr. 9–11, 91054 Erlangen, Germany; 2 Institute of Occupational, Social and Environmental Medicine, University Medical Center, Johannes Gutenberg-University, Obere Zahlbacher Strasse 67, 55131 Mainz, Germany

**Keywords:** Biomonitoring, Firefighters, Urinary metabolome, Untargeted metabolomics, Tryptophan metabolism, Catecholamine metabolism

## Abstract

Exposure biomonitoring is limited to quantifying the internal dose of specific contaminants. However, it fails to capture the complex total biological burden comprising chemical mixtures (such as fire emissions) and physical and psychological stress. Effect biomonitoring, particularly using untargeted metabolomics, is required to comprehensively assess the systemic health effects of these complex exposures. This pilot study aimed to identify metabolic changes by analyzing urine samples collected from two individual firefighters before and after two controlled training scenarios. One scenario measured physical stress (using respiratory protection only), while the other measured additional smoke exposure (using a controlled fire). Samples were derived from an established exposure biomonitoring study which confirmed relevant additional exposure for 10 polycyclic aromatic hydrocarbons (PAHs) in the fire scenario despite protective gear. Urinary metabolites were extracted and analyzed using untargeted gas chromatography-mass spectrometry, followed by data analysis using MetaboAnalyst and R software. 79 human metabolites were identified. Principal component analysis showed a clear separation of the metabolic profiles. A t-test identified four metabolites specifically and significantly regulated by fire exposure: a decrease in catechol and 3-hydroxyphenylacetic acid, and an increase in 5-hydroxy-L-tryptophan and serotonin. Pathway analysis confirmed that the tryptophan and catecholamine pathways were primarily impacted. The observed metabolic changes indicate a significant systemic stress response and biological effect caused by fire exposure. Although metabolic changes indicate biological effects, the study conditions do not allow for distinguishing between toxicological and non-toxicological stressors (i.e. heat). The study demonstrates the potential of urinary metabolomics as a non-invasive method for supplementing effect biomonitoring for firefighters.

**Supplementary Information:**

The online version contains supplementary material available at 10.1007/s00204-026-04397-w.

## Introduction

In the fields of occupational medicine and environmental toxicology, exposure biomonitoring represents the established standard for assessing the internal dose of hazardous substances, typically by quantifying specific, predefined contamintants or their metabolites in biological matrices such as urine. While this single-parameter approach successfully provides an estimate of the individual internal dose, it is limited in its ability to address the whole complexity of exposure scenarios (Hopf et al. 2025). Humans are frequently exposed not just to single toxicants, but to intricate chemical mixtures. Furthermore, the true measure of adverse health risk must incorporate the total biological burden on an individual, which extends beyond chemical agents to include systemic physiological stressors like heat and physical or psychological strain. Consequently, a methodology is required that can capture the collective biological impact resulting from the complex interplay of both chemical mixtures and accompanying stress factors, effects that are inherently invisible to exposure biomonitoring.

To address this critical gap and to move beyond the limitations of exposure biomonitoring, effect biomonitoring is becoming increasingly necessary. By conducting a comprehensive, non-invasive analysis of the metabolome—the collection of small-molecule metabolites, ideally in urine—untargeted metabolomics allows for the identification of systemic changes in metabolic pathways. These metabolic perturbations function as sensitive, holistic indicators of the biological effect, thus complementing exposure biomonitoring. However, effect biomonitoring must be interpreted within the context of known exposure data to ensure scientific rigor.

In fire fighting operations, firefighters face a complex total biological burden as they are exposed to a wide range of hazardous substances, such as polycyclic aromatic hydrocarbons (PAHs), as well as physical and psychological stress. Prior research employing conventional biomarkers of effect - such as those related to oxidative stress, inflammation, vascular damage, and tissue injury - has repeatedly proven an association between fire and/or heat exposure and significant biological alterations in firefighters (Barros et al. 2021). However, most of these reports rely on cross-sectional designs, which fundamentally limit the ability to fully characterize risk factors and establish a clear association with the development of work-related diseases. Similarly, metabolomic studies on firefighters, such as those by the working group of Burgess, have demonstrated significant acute metabolic alterations post-fire exposure (Furlong et al. 2023; Liu et al. 2025). Other studies, such as the work by Rotander et al., have instead focused on chronic, non-acute exposures, like per- and polyfluoroalkyl substances (PFAS) (Rotander et al. 2024). Nevertheless, the exposure studies were typically based on real structural fire incidents, a scenario which is scientifically limited by the highly variable and often unquantifiable nature of the chemical exposure, making it difficult to precisely attribute specific metabolic signatures to toxicological mechanisms. Furthermore, sampling time may play a crucial role for investigating exposure and potentially related effects with scheduled sampling often being difficult when studying emergency responders on duty.

Therefore, the aim of the present pilot study was to investigate the biological effect of fire exposure by applying untargeted metabolomics to urine samples derived from a study by Rossbach et al. (Rossbach et al. 2020). Rossbach et al. used a highly controlled single-arm experimental design involving firefighting instructors exposed during standardized live-fire training, ensuring that fire exposure was the primary independent variable. The samples are already characterized by quantitative data on PAH uptake through exposure biomonitoring, providing the essential contextual knowledge necessary for the robust evaluation of the resulting metabolic profiles. By applying untargeted metabolomics to these samples, we seek to identify metabolic signatures that reflect the comprehensive biological effect of a defined fire exposure, including its chemical but also its physical and psychological stress components, thereby complementing existing biomonitoring parameters in occupational medicine with metabolic profiles.

## Materials and methods

### Chemicals and reagents

All chemicals were purchased at Merck KGaA (Darmstadt, Germany) at a purity suitable for gas chromatography.

(Acetonitrile ≥ 99.9%, suitable for LC/MS, LiChrosolv, HPLC Plus, for HPLC, GC, and residue analysis, ≥ 99.9%;^®^; N-methyl-N-trimethylsilyltrifluoracetamid, derivatization grade LiChropur™, ≥ 98.5%; chlorotrimethylsilan, puriss., ≥ 99.0%; methoxyamin-hydrochlorid, derivatization grade, LiChropur™, 97.5-102.5%)

### Subjects

For this pilot study, we utilized samples from two male non-smoking firefighting instructors who participated in a larger controlled biomonitoring investigation on PAH exposure during live fire training (Rossbach et al. 2020). The participants were professionally qualified, routinely checked by an occupational physician, and utilized self-contained breathing apparatus during the fire exposure sessions. Detailed information regarding their health status, age distribution, exclusion criteria (e.g., non-smoking status, no tattoos, no beard), ethical approval (Rhineland-Palatinate State Chamber of Physicians, ref. no. 2018 − 13952-clinical research), and informed consent is described in the main study (Rossbach et al. 2020).

### Fire exposure protocol

The controlled fire exposure was performed in a dedicated, contained live fire training unit at the Frankfurt/Main fire service training center, adhering to a well-defined protocol (Rossbach et al. 2020).

The standardized fuel source was virgin uncoated chipboards, consisting primarily of wood chips (85% total) and additives, including binders based on urea resins, melamine urea resins, or diphenylmethane diisocyanate (up to 10% maximum). Participants were exposed to combustion products for 30–40 min while wearing full personal protective equipment, including self-contained breathing apparatus. The total session duration was 1.5–2.0 h (mean 1.75 h), which included preparatory work and subsequent undressing and body cleaning.

### Sample collection

Each participant was subjected to two distinct exposure scenarios: one involving fire exposure (RDA) and one involving physical exertion only (AS), serving as active control for metabolic stress (cross-over design).

Urine collection for both scenarios spanned approximately 24 h, starting the evening prior to the exercise. Following the protocol described by Rossbach et al., participants collected every individual urine void in separate vessels at eight to nine predefined time points (Rossbach et al. 2020). This period was segmented into two volume-weighted pooled samples per session: a pre-exposure sample (comprising all voids after going to bed on the previous evening until the start of the exercise at ~ 10:00 AM) and a post-exposure sample (comprising all voids from the end of the 1.75 h training session until the last void before bedtime). This standardized pooling of individual voids using volume-weighted aliquots ensures that the samples represent the integrated internal exposure and the toxicokinetic recovery phase, while minimizing the impact of short-term fluctuations. This sampling design results in the pre-exposure sample consisting mainly of night urine, while the post-exposure sample consists mainly of day urine (Supplementary Information Figure S1). We addressed this inherent temporal confounder by comparing the fire exposure (RDA) outcome to the active control (AS) outcome.

In total, eight biological pooled urine samples were obtained (2 participants × 2 sessions × 2 timepoints). For quality control, these eight samples were processed in duplicate, and an additional pooled Quality Control (QC) sample was generated and processed once, resulting in 17 probes for GC-MS analysis.

### Sample preparation

Following sample preparation 300 µl of -20 °C acetonitrile was added to 100 µl urine, shaken for one minute and centrifuged at 18000rcf, 5 °C for 5 min. The organic phase was dried using a speedvac vacuum dryer. The residue was resuspended in 100 µl of dichlormethane and the previous drying procedure repeated.

For derivatization purposes, the dried fractions were resuspended with 50 µL of 20 mg/mL methoxyamine hydrochloride in pyridine, sonicated for 2 min and then incubated at 50 °C for 90 min. This was followed by derivatization with 50 µL of N-methyl-N-(trimethylsilyl)-trifluoroacetamide (MSTFA) containing 1% trimethylchlorosilane (TMCS) at 40 °C for 1 h and frozen at -80 °C until measurement (Olivier et al. 2023).

### GC-MS analysis

Derivatized metabolites were analyzed by gas chromatography-mass spectrometry (GC-MS) using an Agilent 8890 GC coupled to an Agilent 5977B quadrupole MS, as previously described (Kersch et al. 2025). Briefly, a nonpolar-low polar OPTIMA 5 MS column (30 m x 0.32 mm i.d., 0.25 μm film thickness) was employed. Two microliters of sample were injected in splitless mode into the 250 °C inlet, with helium as the carrier gas (1.5 mL/min). The oven temperature program began with a 10-minute hold at 80 °C, ramped at 5 °C/min to 330 °C, and concluded with a final 10-minute hold.

### Data processing and statistical analysis

Raw mass spectrometry (GC-MS) data underwent initial processing, including peak deconvolution and metabolite annotation, utilizing R-based tools supported by the annotated Golm Metabolome Database. The processed data were subsequently normalized, log10 transformed, and pareto scaled prior to statistical analysis. Given the highly limited sample size (*n* = 2 per group), we treated each sample as an independent replicate rather than employing a paired analysis (which would have been statistically more powerful but precluded by the small sample size) to ensure sufficient degrees of freedom for robust statistical treatment of the overall treatment effect. As a pilot study, all subsequent statistical results, including univariate and multivariate findings, are mainly descriptive and hypothesis-generating. All core statistical methods, encompassing both univariate comparisons (t-tests and ANOVA) and multivariate analyses [principal component analysis (PCA), partial least squares-discriminant Analysis (PLS-DA)] were performed using the web-based platform MetaboAnalyst 6.0. PLS-DA was used specifically for descriptive variable importance in projection (VIP) ranking. To statistically assess the separation between groups, a PERMANOVA was conducted on the PCA data, as this approach is more suitable for significance testing in small-scale pilot studies than predictive PLS-DA modeling (Ruiz-Perez et al. 2020).

For functional and interpretive analysis, pathway analysis (using databases such as RaMP-DB) and network analysis were conducted. To exploratively assess the biomarker potential of individual and combined metabolites, a receiver operating characteristic (ROC) curve analysis was performed (MetaboAnalyst 6.0).

## Results

To investigate the metabolic impact of firefighting on the human body, we performed an untargeted GC-MS analysis of urine samples collected from a controlled single-arm exposure study. We sought to determine whether additional fire exposure during a controlled training session would lead to significant changes in the metabolic profiles of firefighters, as compared to a similar session without fire exposure. This question was addressed by analyzing urine samples from two distinct groups: the RDA group (with fire exposure) and the AS group (without). Using samples from two study participants, each urine sample was considered a biological replicate as the samples were collected on different days. While a paired analysis would have been statistically more powerful, the small sample size precluded this approach. Therefore, we treated each sample as an independent replicate to perform a robust statistical analysis of the treatment effect.

From the untargeted analysis, we identified a total of 79 human-relevant metabolites with a match factor greater than 0.9 against the Golm Metabolome Database (GMD). A complete list of these identified compounds is provided in Supplementary Information Table S1.

To illustrate the overall variability within the dataset, we performed a heatmap analysis using log2(FC) values (Supplementary Information Fig. S2). This analysis showed that the largest differences were driven by inter-individual variability among the participants. While a treatment-related effect (RDA vs. AS) was also observed, this high biological variability is a common phenomenon in studies with small sample sizes.

To specifically address our scientific question regarding the effect of firefighting exposure on metabolic profiles, we therefore employed subsequent multivariate and statistical analyses.

### Multivariate analysis reveals a distinct metabolic signature

**Fig. 1:**
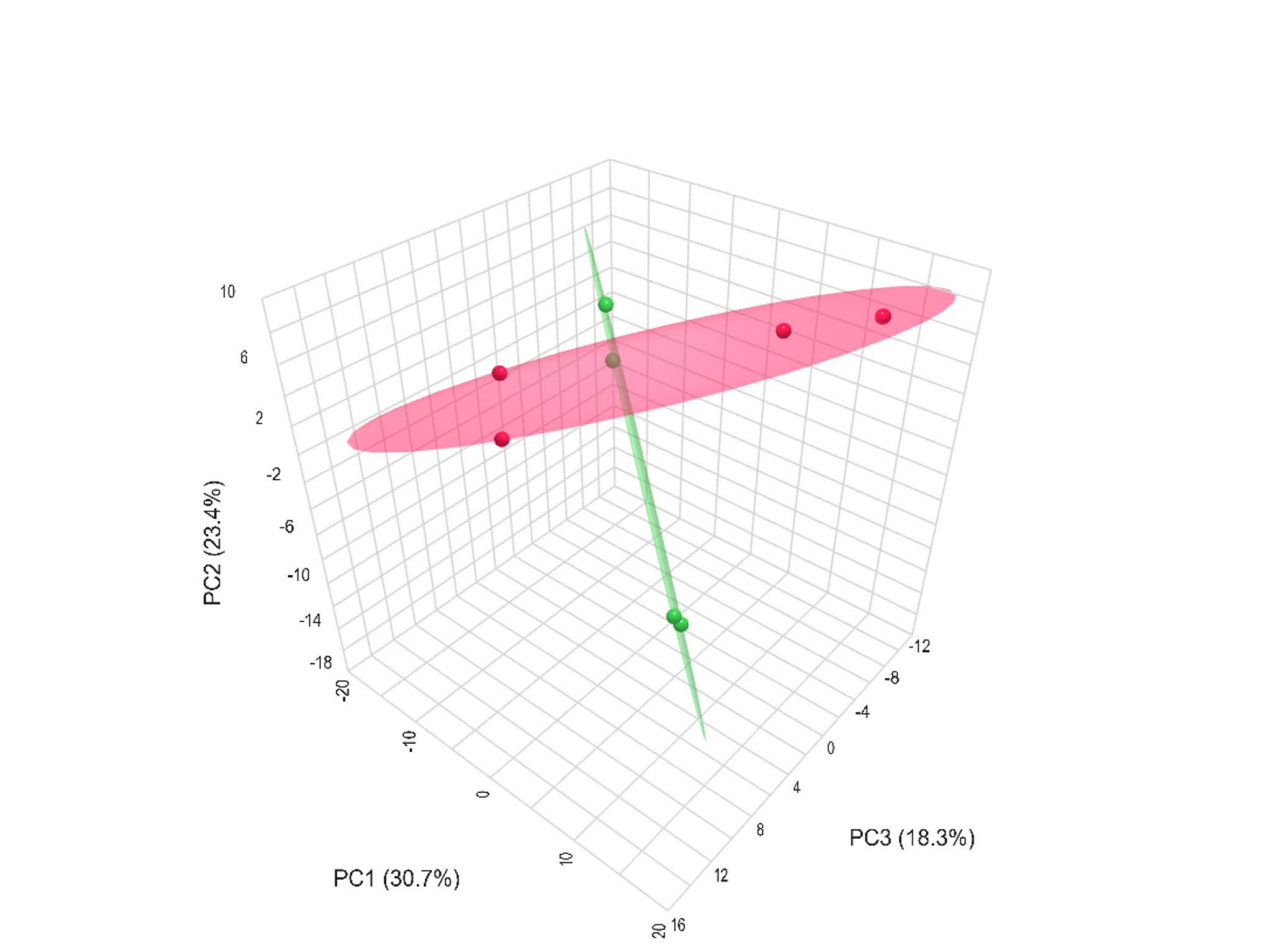
3D PCA analysis of the metabolic profiles. The scores plot visualizes the separation of treatment groups based on a principal component analysis (PCA). Each data point represents a urine sample (biological replicate)

Principal component analysis (PCA) was performed to visualize the overall metabolic profiles and identify the primary sources of variation within the dataset. A three-dimensional PCA scores plot (Fig. 1) of the log2(FC) values shows a separation trend of the fire exposure group (RDA) from the AS group, which performed training without fire exposure. The first three principal components collectively accounted for 88.3% of the total variance, indicating that the main patterns in the data are robustly captured.

While visual clustering in small datasets can be subtle, the PERMANOVA analysis confirmed a strong biological effect of the fire exposure on the urinary metabolome (R-squared = 40.9%, p-value = 0.054). While the p-value was just above 0.05, the substantial effect size is highly notable and points to a biologically relevant distinction between the groups. The slight discrepancy in statistical significance can be attributed to the low number of participants and the inherent biological variability.

To identify the metabolites that most effectively discriminate between the groups, we conducted a partial least squares-discriminant analysis (PLS-DA) and used the variable importance in projection (VIP) scores to rank the metabolites according to their contribution to the group separation. As shown in the VIP scores plot (Fig. S3), several metabolites were identified as key discriminators. The most influential metabolites included catechol, 3-hydroxyphenylacetic acid, 5-hydroxy-L-tryptophan, serotonin and shikimic acid. These results confirm that a specific set of metabolites is primarily responsible for the distinct metabolic signature observed in the RDA group.

Subsequent t-tests were used to pinpoint individual metabolites that were significantly different between the RDA and AS groups. The log2 fold change between the ‘before’ and ‘after’ samples was calculated, and a false discovery rate (FDR) adjusted p-value threshold of 0.05 was applied to control for multiple comparisons. Compared to the AS group, additional fire exposure resulted in significantly altered levels of four metabolites in the RDA group: decreased catechol and 3-hydroxyphenylacetic acid, and increased 5-hydroxy-L-tryptophan and serotonin (Table S2).

These robust statistical findings provide a strong foundation for exploring the biological consequences of fire exposure. The significant changes in key metabolites strongly suggest a perturbation of critical metabolic pathways. Based on these statistical findings, we proceeded with a more detailed biological interpretation of the regulated metabolites.

### Volcano plot analysis: identifying key regulated metabolites

The volcano plot serves as a visual tool that integrates the statistical significance (p-value) and the magnitude of change (fold change) into a single, comprehensive overview. This analysis enabled us to pinpoint the metabolites that were both statistically significant and biologically meaningful perspectives not fully captured by VIP scores or t-tests alone.

Downregulated metabolites in the RDA group (Tables S3):

- Catechol and 3-hydroxyphenylacetic acid: Consistent with the t-test results, the volcano plot also shows a very strong downregulation of these two metabolites following fire exposure. 3-Hydroxyphenylacetic acid is a metabolite of phenylalanine and tyrosine catabolism and can also originate from microbial metabolism. Its severe reduction points to profound changes in these pathways.
- Glycolic acid and shikimic acid were also strongly downregulated, but their p-values were not statistically significant. Glycolic acid is a metabolite involved in the glyoxylate and amino acid metabolism, while shikimic acid, a key intermediate in the shikimate pathway, is often indicative of human gut microbiota activity and is directly linked to the tryptophan and tyrosine metabolism.
- Aconitic acid (TCA cycle), phytosphingosine (cell membranes), indole-3-acetaldehyde (tryptophan metabolism), pipecolic acid (lysine catabolism, oxidative stress protection (Natarajan et al. 2017), and homovanillic acid (dopamine degradation) were also downregulated by fire exposure, though some changes were not statistically significant.

Upregulated metabolites in the RDA group:

- 5-Hydroxy-L-tryptophan and serotonin were both strongly upregulated and highly significant, indicating a major disturbance in the tryptophan-serotonin metabolic pathway.
- Indolelactate (a tryptophan metabolite), L-tyrosine (a precursor for thyroid hormones and catecholamines), and norvaline (an arginase inhibitor and a factor in NO production (Chang et al. 1998; Gilinsky et al. 2020)) were upregulated, though these changes were not always statistically significant.

### Correlation network analysis: uncovering functional relationships

To move beyond a list of individual metabolites and understand how they interact as a system, we performed a correlation network analysis. This step allowed us to visualize the coordinated metabolic response to the exposure and understand the regulation within the RDA group. This analysis reveals the “hubs” or “gatekeepers” of the metabolic network—metabolites that are highly connected to others and thus play a central, regulatory role. It also identifies specific, strong correlations that may indicate functional coupling between metabolites under a given condition.

In the sparse RDA network, the key metabolites were galactose, which had the highest connectivity, and the catecholamine-related metabolites p-hydroxymandelic acid and DOPA (Fig. S4). The central position and high relevance of these metabolites in this stringent network suggest they are the primary regulators of the metabolic changes within the RDA group. The overall high relevance of these metabolites in this network points to carbohydrate and catecholamine metabolism as the primary and most directly impacted pathways in response to additional fire exposure.

### Pathway analysis: mapping metabolites to biological processes

Complementary to the group-specific network analysis, we performed a pathway analysis to investigate the general regulatory changes across all samples, independent of their group assignment. The analysis, which was conducted using metabolites with a VIP score > 1 and log2(FC) values, confirmed that the tryptophan metabolism pathway was the most significantly affected, aligning perfectly with the strong changes observed in 5-hydroxy-L-tryptophan and serotonin. The analysis also highlighted significant changes in tyrosine metabolism (pathway for catecholamines), sphingolipid metabolism (related to cellular stress and membranes), and core energy metabolism pathways such as the TCA cycle and fatty acid degradation (Table 1).

**Table 1:** Statistically significant enriched metabolic pathways (FDR < 0.05)

Pathways	Total Cmpd	Hits	Raw *p*	LOG10(*p*)	Holm adjust	FDR	Impact
Tryptophan metabolism	41	3	2.06E-05	4.7	0.0003	0.000	0.2498
Sphingolipid metabolism	32	2	0.004	2.4	0.0578	0.031	0.0796
Fatty acid degradation	39	1	0.014	1.8	0.1998	0.048	0
Citrate cycle (TCA cycle)	20	1	0.019	1.7	0.2428	0.048	0.05
Glyoxylate and dicarboxylate metabolism	32	2	0.021	1.7	0.2533	0.048	0.03
Tyrosine metabolism	42	3	0.022	1.7	0.2533	0.048	0.25
Lysine degradation	30	1	0.025	1.6	0.2533	0.048	0
Phenylalanine, tyrosine and tryptophan biosynthesis	4	1	0.030	1.5	0.2685	0.048	0.5
Ubiquinone and other terpenoid-quinone biosynthesis	19	1	0.030	1.5	0.2685	0.048	0
Phenylalanine metabolism	8	1	0.030	1.5	0.2685	0.048	0

The RaMP-DB (Relational database of Metabolomics Pathways DataBase) analysis (Braisted et al. 2023) further refined these findings by specifically pinpointing serotonin-related pathways, including its biosynthesis, clearance, and receptor activity, providing highly specific evidence for a disruption in the serotonergic system (Table S4).

### Biomarker analysis: identification of a distinct metabolic signature

To explore the potential of individual metabolites to serve as effective classifiers, we conducted a receiver operating characteristic (ROC) curve analysis on the post-exposure samples. It is essential to note that due to the very small sample size of this pilot study, the calculated area under the curve (AUC) values are descriptive and exploratory only and cannot be considered validated biomarkers. The results are strictly hypothesis-generating.

Our univariate ROC analysis revealed a remarkable finding: a panel of 12 metabolites achieved a perfect area under the ROC Curve (AUC = 1.0), indicating their ability to separate the RDA and AS groups without any misclassification. These discriminators included catechol, 3-hydroxyphenylacetic acid, shikimic acid, and indolelactate, all of which have been highlighted in our previous analyses as key differentiating metabolites (Table S5).

Building on these findings, we performed an exploratory multivariate ROC analysis using a linear support vector machine (SVM) model with Monte-Carlo cross-validation to identify the most informative metabolites for classification. While the model achieved an AUC approaching 1.0—indicating a strong separation between the few available samples—it is imperative to state that this performance reflects potential overfitting due to the sample size and must be interpreted strictly as hypothesis-generating. The analysis successfully identified a small, prioritized panel of metabolites (Table S6) that are the strongest candidates for distinguishing the two exposure states and require validation in a larger, independent cohort.

The most important metabolites in this multivariate model were identified by their ranking frequency, which measures how consistently a metabolite is selected as a top feature across all cross-validation iterations. Shikimic acid emerged as the most consistent top biomarker, followed by D-2-hydroxyglutaric acid and hippuric acid (0.34). Notably, catechol, which was a perfect discriminator in the univariate analysis, also maintained a solid position in the multivariate model. These results confirm that while several metabolites can individually separate the groups, the multivariate approach provides a more robust and stable set of biomarkers that consistently characterize the metabolic signature of fire exposure.

### Integration of key findings: a central role for tryptophan and tyrosine metabolism

**Fig. 2:**
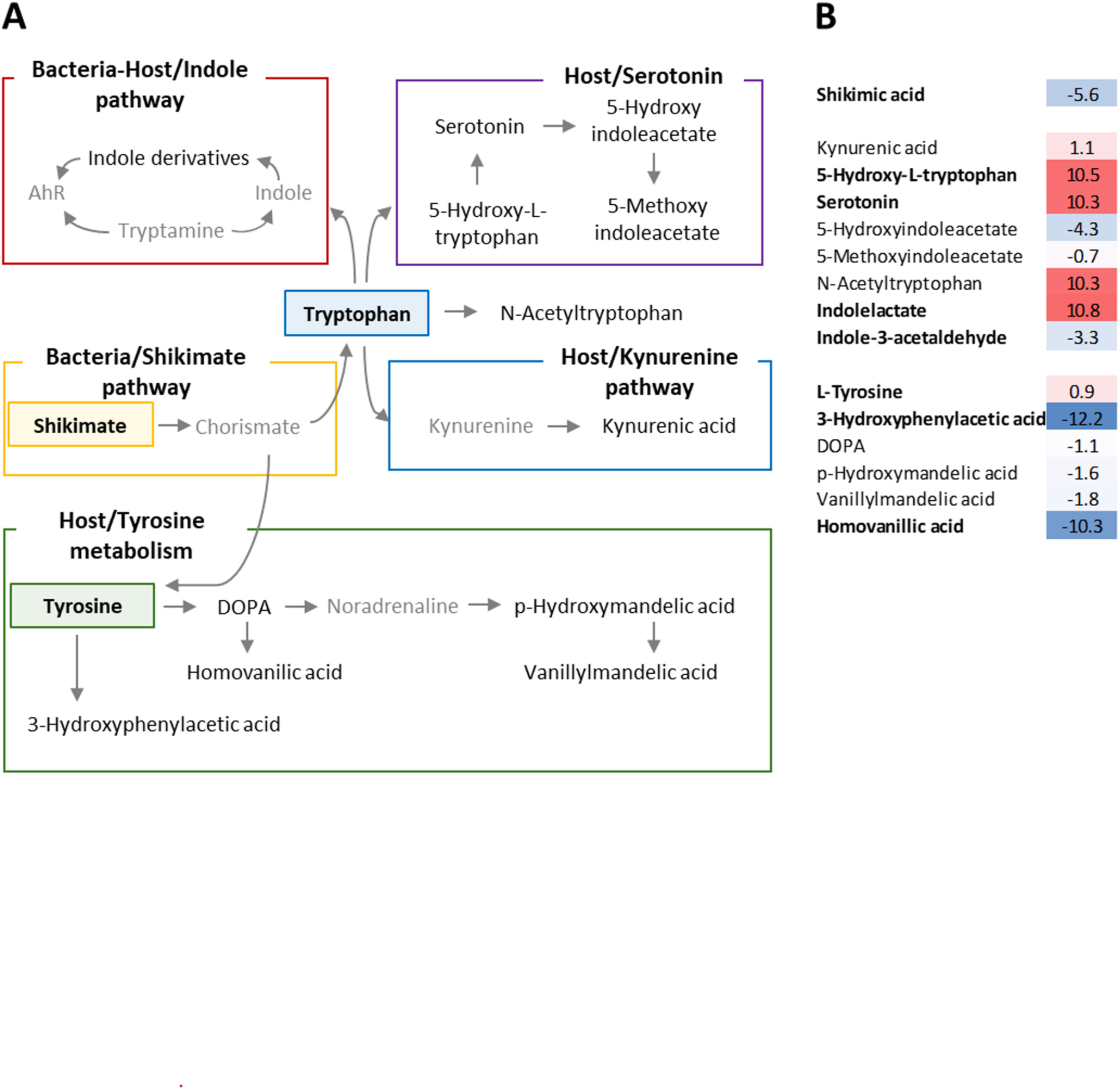
Overview of the major metabolic pathways affected by exposure conditions and the corresponding fold changes of key metabolites. **A** A schematic representation of the metabolic pathways. Metabolites identified in our analysis are shown in bold, while those not identified are in gray. **B** A heatmap of the log2 fold changes for identified metabolites. Metabolites shown in bold were found to be statistically significant in the volcano plot analysis

To consolidate the findings from our multivariate, network, and pathway analyses, we integrated the most significant metabolites into an overview as illustrated in Fig. 2. This visual summary highlights the central role of key metabolic pathways in the response to fire exposure.

- Pathway-level perturbation: Consistent across multiple analyses, the tryptophan metabolism pathway was identified as the most significantly affected biological process. This is strongly supported by the upregulation of its downstream products, 5-hydroxy-L-tryptophan and serotonin (Figure B).
- Key biomarkers: The biomarker analysis further underscored the importance of this pathway and its connections to others. Metabolites from the shikimate pathway (shikimic acid) and the tyrosine metabolism (p-hydroxymandelic acid, DOPA) were among the most effective discriminators in separating the RDA and AS groups.
- Network-level regulation: The correlation network analysis provided a group-specific insight into the regulatory dynamics. It revealed that in the RDA group, metabolites related to tyrosine metabolism—specifically DOPA and p-hydroxymandelic acid—played a central, regulatory role within the network. This finding indicates that while multiple pathways are perturbed, the catecholamine-related response is an important driver of the metabolic signature observed in firefighters after fire exposure.

## Discussion

### Integration of exposure biomonitoring and metabolic effect analysis

The primary objective of this study was to leverage a strictly controlled experimental design and untargeted urinary metabolomics to identify the specific metabolic impact of acute fire exposure compared to training in protective gear. A key methodological strength lies in the synergy between our untargeted metabolomics approach (effect biomonitoring) and the concurrent, targeted exposure biomonitoring from the underlying Rossbach et al. study, from which our samples were derived (Rossbach et al. 2020). The Rossbach study quantified the internal dose of specific toxins, importantly showing a significant increase in all 10 measured mono-hydroxylated PAH metabolites (i.e. naphthalene, fluorene, phenanthrene, and pyrene). While exposure biomonitoring is typically limited to measuring single parameters, our metabolomics approach enables us to capture the comprehensive biological effect of total burden parameters. This aligns with current toxicological frameworks emphasizing that untargeted omics-related tools are paramount for understanding the pathways associated with adverse outcomes when subjects are exposed to complex chemical mixtures (Hernandez et al. 2019). The critical consequence of this unique data integration is a robust, causal framework: the confirmed, quantified internal dose of toxicants (Rossbach data) provides the necessary basis to attribute the observed systemic metabolic shifts (our metabolomics data) directly to the acute exposure, setting our findings apart from studies that lack this established quantitative exposure anchor.

### Metabolic perturbation of aromatic amino acids: the role of the host-microbiome axis

A key finding of our study is the significant perturbation of the tryptophan and tyrosine metabolism pathways, an observation partially consistent with the findings of Liu et al. (2025). We identified the metabolite shikimic acid—a known precursor for the synthesis of the aromatic amino acids phenylalanine, tyrosine, and tryptophan (Kumariya et al. 2024; Tzin et al.)—as a central starting point in our metabolic analysis. Our findings demonstrated a strong regulation of the tryptophan pathway, specifically showing a significant up-regulation of indole acetates (in the indole pathway), along with serotonin and 5-hydroxy-L-tryptophan (in the serotonin pathway). In contrast, the tyrosine metabolism showed a significant down-regulation of metabolites such as 3-hydroxyphenylacetic acid and homovanillic acid.

Interestingly, the microbial-derived nature of shikimic acid and parts of the indole pathway strongly suggests a central link between the acute exposure and the host-microbiome axis. While the gut microbiome is often the primary focus in metabolomic studies, the specific exposure route in this study must be considered: participants used self-contained breathing apparatus, making dermal uptake the dominant route for PAHs.

This raises two distinct possibilities for the observed regulation of shikimic acid. On one hand, the significant decrease in shikimic acid may reflect a localized metabolic perturbation of the skin microbiota (e.g., Corynebacterium (Callewaert et al. 2013; Sheng et al. 2023) in direct response to the dermal PAH load. PAHs are known to exert selective pressure on microbial communities and can interfere with bacterial metabolic pathways (Leung et al. 2023). On the other hand, if the metabolic shift is part of a more generalized, non-specific systemic stress response, the gut microbiome could also be a significant contributor. Systemic physiological stress, such as heat or physical exertion, can alter gut permeability and microbial activity, potentially impacting the shikimate pathway regardless of the direct entry route of toxins.

Crucially, the products of this pathway—including indole derivatives—are established ligands of the AhR (Kumar et al. 2021). Since PAHs also act as potent AhR ligands, a simultaneous activation by both microbial metabolites and exogenous chemical toxins could lead to an amplification of the AhR-mediated response. This synergism, whether triggered specifically by dermal PAH-microbe interactions or more broadly via the gut-microbiome axis, could significantly increase the risk of adverse health outcomes, such as carcinogenesis, for firefighters.

### Network analysis confirms activation of the acute physiological stress axis

For a complete biological understanding, it is crucial not only to demonstrate discrimination between groups but also to explore the regulatory nature of the perturbed metabolites within a specific exposure context. We therefore performed a subsequent network analysis focused on the fire-exposed (RDA) group. This analysis provided group-specific insight into the regulatory dynamics, revealing that metabolites related to tyrosine metabolism—specifically DOPA and p-hydroxymandelic acid—acted as central regulatory hubs within the network. This finding is highly significant, especially when viewed against the downregulation of the catabolites: The concurrent central importance of catecholamine precursors/intermediates (DOPA, p-hydroxymandelic acid) and the suppression of the final breakdown products (3-hydroxyphenylacetic acid, homovanillic acid) suggests a strong, coordinated metabolic shunting. This pattern implies that the tyrosine pool is aggressively redirected to fuel the acute catecholamine-related stress response (i.e., fight-or-flight hormones (Romero 2024) confirming that this stress axis is not merely an incidental finding, but rather a central driver of the distinct metabolic signature observed in firefighters. This conclusion is strongly supported by the simultaneous and significant upregulation of serotonin and its precursor 5-hydroxy-L-tryptophan in the tryptophan pathway. This finding is key, as the kynurenine pathway—which typically becomes activated under chronic stress and inflammation by enzymes like tryptophan 2,3 dioxygenase to deplete tryptophan reserves—was not upregulated. Thus, the observed serotonin increase is consistent with a rapid, physiological neuroendocrine response that prioritizes acute survival functions over the resource shunting characteristic of chronic stress (Chaouloff et al. 1999; Kang et al. 2023; La Torre et al. 2021).

Given the strictly controlled cross-over study design, where the exposed group differs only by the fire exposure itself, it remains unclear which specific component primarily triggers this potent stress response: Is it attributable to the psychological/visual stimulus (a conditioned reaction), thermal stress (heat), or the quantified chemical load (e.g., elevated PAHs)? Elucidating this complex etiology should be the subject of future investigations. In essence, the network structure highlights the physiological stress response as a primary coordinating factor of the systemic metabolic shift.

### Effect biomonitoring: identifying a coordinated metabolite panel for acute stress

Together, these results indicated that the metabolic response to fire exposure is a coordinated systemic perturbation driven by a few central pathways, particularly tryptophan and tyrosine metabolism. The implication of our results lies in the identification of potential effect biomarkers that reflect this robust metabolic perturbation. A panel of metabolites, such as shikimic acid, D-2-hydroxyglutarate, and hippuric acid, which emerged as consistent classifiers in the multivariate analysis, are not just indicators of chemical exposure, but rather reflect the coordinated systemic metabolic burden associated with the acute firefighting event. As such, these identified biomarkers, derived from effect biomonitoring, could be utilized in the future to reliably assess the physiological impact during emergency operations.

### Study limitations and future perspectives

Despite the methodological rigor, the results must be interpreted within the context of our study limitations. Foremost, this is a pilot study with a highly restricted sample size. This inherent limitation means that all statistical outcomes are mainly descriptive and hypothesis-generating, precluding definitive conclusions regarding biomarker validation or statistical significance. Future, larger-scale studies are warranted to validate the identified metabolite panels and to confirm the robustness of the observed pathway perturbations. Such studies are necessary to further explore ‘real-life risk’ scenarios, where the joint impact of physical stressors (e.g., noise or heat) and toxic gases can significantly potentiate non-specific systemic responses (Onishchenko et al. 2023). Furthermore, investigating the modulation of adaptive cellular signaling—such as the HIF-1α pathway, which orchestrates responses to oxidative stress and toxic insults (Aschner et al. 2023)—could provide deeper mechanistic insights into the integrated biological burden observed in our pilot data.

## Conclusion

This pilot study, while limited by its restricted sample size and thus strictly hypothesis-generating, established a critical methodological framework. Our results reveal significant perturbations in the tryptophan and tyrosine metabolism pathways, suggesting a central role for the acute catecholamine-related stress response, evidenced by a pattern of metabolic shunting. This work demonstrates the distinct power of effect biomonitoring to assess the total biological burden of complex toxic mixtures. The identified panel of classifying metabolites are promising candidates for further validation as robust, non-invasive biomarkers in larger cohorts for occupational health surveillance.

## Supplementary Information

Below is the link to the electronic supplementary material.

Supplementary Material 1

## Data Availability

Data are available from the corresponding author upon request.
