# Supplementary material for: Effect biomonitoring in a controlled firefighting setting: an untargeted metabolomic pilot study
Source: Arch Toxicol. 2026 Apr 25;100(7):3199–209. doi: 10.1007/s00204-026-04397-w (PMC13309430; doi:10.1007/s00204-026-04397-w)
Supplement: Supplementary file 1 — Supplementary Material 1 [file 204_2026_4397_MOESM1_ESM.docx]

**Supplementary Information**

Effect biomonitoring in a controlled firefighting setting: an untargeted metabolomic pilot study.

**Max-Philipp Boehler^a^, Christian Kersch^a^, Bernd Rossbach^b^, Andrea Kaifie^a^, Simone Schmitz-Spanke^a^**

**^a^Institute and Outpatient Clinic of Occupational, Social, and Environmental Medicine, Friedrich-Alexander-University of Erlangen-Nuremberg, Henkestr. 9–11, 91054 Erlangen, Germany**

[maxphilipp.boehler@fau.de](mailto:maxphilipp.boehler@fau.de); [christian.kersch@fau.de](mailto:christian.kersch@fau.de)**;** [andrea.kaifie-pechmann@fau.de](mailto:andrea.kaifie-pechmann@fau.de)**;** [simone.schmitz-spanke@fau.de](mailto:simone.schmitz-spanke@fau.de)

**^b^Institute of Occupational, Social and Environmental Medicine, University Medical Center, Johannes Gutenberg-University, Obere Zahlbacher Strasse 67, 55131 Mainz, Germany**

**Bernd.Rossbach@unimedizin-mainz.de**

**Corresponding Author:**

**Simone Schmitz-Spanke**

**simone.schmitz-spanke@fau.de**

**Institute and Outpatient Clinic of Occupational, Social, and Environmental Medicine, University of Erlangen-Nuremberg, Henkestr. 9-11, 91054 Erlangen, Germany**

**Phone: +49 09131/85-22255**

**ORCID:** 0000-0002-0416-8236

**Table of contens**

[**1.** **Supplementary tables** 3](#_Toc223443132)

[Table S 1: List of all metabolites identified by GC-MS with match factors >0.9 3](#_Toc223443133)

[Table S2: Differentially expressed metabolites between 'before' and 'after' samples 5](#_Toc223443134)

[Table S3: Differentially expressed metabolites with a FC threshold of 1.5 and a raw p-value threshold of 0.1 using volcano plot analysis 5](#_Toc223443135)

[Table S4: Significantly enriched metabolic pathways from RaMP-DB analysis 6](#_Toc223443136)

[Table S5: Univariate ROC analysis of metabolites 11](#_Toc223443137)

[Table S6: Key metabolites from multivariate ROC analysis 11](#_Toc223443138)

[**2.** **Supplementary figures** 13](#_Toc223443139)

[Figure S1: Schematic representation of the study design and 24-hour urine sampling protocol. 13](#_Toc223443140)

[Figure S2: Heatmap of metabolite log2(FC) values 14](#_Toc223443141)

[Figure S3: PLS-DA VIP scores plot. 15](#_Toc223443142)

[Figure S4: Metabolite correlation network for the RDA group 16](#_Toc223443143)

# **Supplementary tables**

Table S 1: List of all metabolites identified by GC-MS with match factors >0.9 according to the Golm Metabolome Database

| **No.** | **Metabolites** |
| --- | --- |
| 1 | (2E)-Decenoyl-ACP |
| 2 | 1,2,3,4-Tetrahydro-2-methyl-b-carboline |
| 3 | 2-Hydroxybutyric acid |
| 4 | 3-hydroxyisovaleric acid |
| 5 | 3-Hydroxyphenylacetic Acid |
| 6 | 5-Hydroxyindoleacetic acid |
| 7 | 5-Hydroxy-L-tryptophan |
| 8 | 5-Methoxyindoleacetate |
| 9 | Aconitic acid |
| 10 | Adenosine |
| 11 | Cadaverine |
| 12 | Catechol |
| 13 | Cholest-5-ene |
| 14 | Cholesterol |
| 15 | Citramalic acid |
| 16 | Citric acid |
| 17 | Creatine |
| 18 | Creatinine |
| 19 | D-2-Hydroxyglutaric acid |
| 20 | Deoxycholic acid |
| 21 | D-Fructose |
| 22 | D-Galactose |
| 23 | D-Glucose |
| 24 | D-Glucuronic acid |
| 25 | DOPA |
| 26 | Ribose |
| 27 | D-Threitol |
| 28 | Fumaric acid |
| 29 | Galactinol |
| 30 | Galactitol |
| 31 | Galactose |
| 32 | Glucose-1-phosphate |
| 33 | Glutamine |
| 34 | Glycerol |
| 35 | Glycine |
| 36 | Glycolic acid |
| 37 | Hippuric acid |
| 38 | Homovanillic acid |
| 39 | Hypoxanthine |
| 40 | Indole-3-Acetaldehyde |
| 41 | Indolelactate |
| 42 | Kynurenic Acid |
| 43 | Lactic acid |
| 44 | L-Arabinose |
| 45 | L-Arabitol |
| 46 | L-Arginine |
| 47 | L-Fucose |
| 48 | L-Lyxonic acid |
| 49 | L-Norleucine |
| 50 | Pipecolic acid |
| 51 | L-Tyrosine |
| 52 | Maleic acid |
| 53 | Mandelic acid |
| 54 | Mannitol |
| 55 | Methionine |
| 56 | MG(0:0/16:0/0:0) |
| 57 | myo-Inositol |
| 58 | N-Acetyl-L-aspartic acid |
| 59 | N-Acetylneuraminic acid |
| 60 | N-Acetyltryptophan (N-acetyl-L-tryptophan) |
| 61 | N-alpha-Acetyllysine |
| 62 | N-methyl-L-glutamic acid |
| 63 | Norvaline |
| 64 | Oleamide |
| 65 | Palmitic acid |
| 66 | Phosphate |
| 67 | p-Hydroxymandelic acid |
| 68 | Phytosphingosine |
| 69 | Pyroglutamic acid |
| 70 | Pyruvic acid |
| 71 | Rhamnose |
| 72 | Serine |
| 73 | Serotonin |
| 74 | Shikimic acid |
| 75 | Sphinganine |
| 76 | Sphingosine |
| 77 | Succinic acid |
| 78 | Uric acid |
| 79 | Vanillylmandelic acid |

Table S2: Differentially expressed metabolites between 'before' and 'after' samples based on log2(fold change) and t-test with a p-value threshold of 0.05 (FDR).

| **Metabolites** | **t.stat** | **p.value** | **-LOG10(p)** | **FDR** |
| --- | --- | --- | --- | --- |
| Catechol | 75.67 | 3.59E-10 | 9.4454 | 2.83E-08 |
| 5-Hydroxy-L-tryptophan | -29.45 | 1.02E-07 | 6.9931 | 4.01E-06 |
| Serotonin | -25.333 | 2.49E-07 | 6.6034 | 6.56E-06 |
| 3-Hydroxyphenylacetic acid | 10.135 | 5.36E-05 | 4.2705 | 0.0010594 |

Table S3: Differentially expressed metabolites with a FC threshold of 1.5 and a raw p-value threshold of 0.1 using volcano plot analysis in MetaboAnalyst 6.0

| **Metabolites** | **FC** | **log2(FC)** | **raw.pval** | **-LOG10(p)** |
| --- | --- | --- | --- | --- |
| Catechol | 0.00011011 | -13.149 | 3.59E-10 | 9.4454 |
| 5-Hydroxy-L-tryptophan | 1431.5 | 10.483 | 1.02E-07 | 6.9931 |
| Serotonin | 1280.2 | 10.322 | 0.22856 | 6.6034 |
| 3-Hydroxyphenylacetic acid | 0.00021983 | -12.151 | 5.36E-05 | 4.2705 |
| Glycolic acid | 0.00044921 | -11.12 | 0.88012 | 1.8456 |
| Shikimic acid | 0.021177 | -5.5613 | 0.8068 | 1.8117 |
| Aconitic acid | 0.080029 | -3.6433 | 0.018676 | 1.7287 |
| Phytosphingosine | 0.00032111 | -11.605 | 0.46527 | 1.6987 |
| Indole-3-acetaldehyde | 0.10097 | -3.308 | 0.065251 | 1.637 |
| Pipecolic acid | 0.00045173 | -11.112 | 0.14256 | 1.6041 |
| L-Tyrosine | 1.8675 | 0.9011 | 0.16077 | 1.5253 |
| Indolelactate | 1803.7 | 10.817 | 0.11853 | 1.5147 |
| Homovanillic acid | 0.00077815 | -10.328 | 0.014268 | 1.1854 |
| Norvaline | 8.0191 | 3.0034 | 0.57954 | 1.0272 |

Table S4: Significantly enriched metabolic pathways from RaMP-DB analysis (FDR < 0.05)

| **Pathways** | **Total Cmpd** | **Hits** | **Statistic Q** | **Expected Q** | **Raw p** | **Holm p** | **FDR** |
| --- | --- | --- | --- | --- | --- | --- | --- |
| Serotonin and melatonin biosynthesis | 15 | 2 | 99.197 | 14.286 | 7.43E-08 | 2.74E-05 | 1.04E-06 |
| G alpha (i) signalling events | 108 | 1 | 99.074 | 14.286 | 2.49E-07 | 9.15E-05 | 1.04E-06 |
| Phase I - Functionalization of compounds | 189 | 1 | 99.074 | 14.286 | 2.49E-07 | 9.15E-05 | 1.04E-06 |
| Metabolic disorders of biological oxidation enzymes | 51 | 1 | 99.074 | 14.286 | 2.49E-07 | 9.15E-05 | 1.04E-06 |
| Hemostasis | 82 | 1 | 99.074 | 14.286 | 2.49E-07 | 9.15E-05 | 1.04E-06 |
| G alpha (s) signalling events | 30 | 1 | 99.074 | 14.286 | 2.49E-07 | 9.15E-05 | 1.04E-06 |
| Amine Oxidase reactions | 18 | 1 | 99.074 | 14.286 | 2.49E-07 | 9.15E-05 | 1.04E-06 |
| Biogenic amines are oxidatively deaminated to aldehydes by MAOA and MAOB | 11 | 1 | 99.074 | 14.286 | 2.49E-07 | 9.15E-05 | 1.04E-06 |
| Organic cation transport | 27 | 1 | 99.074 | 14.286 | 2.49E-07 | 9.15E-05 | 1.04E-06 |
| Neurotransmitter receptors and postsynaptic signal transmission | 25 | 1 | 99.074 | 14.286 | 2.49E-07 | 9.15E-05 | 1.04E-06 |
| Platelet activation, signaling and aggregation | 32 | 1 | 99.074 | 14.286 | 2.49E-07 | 9.15E-05 | 1.04E-06 |
| Platelet degranulation | 11 | 1 | 99.074 | 14.286 | 2.49E-07 | 9.15E-05 | 1.04E-06 |
| Response to elevated platelet cytosolic Ca2+ | 13 | 1 | 99.074 | 14.286 | 2.49E-07 | 9.15E-05 | 1.04E-06 |
| Serotonin clearance from the synaptic cleft | 12 | 1 | 99.074 | 14.286 | 2.49E-07 | 9.15E-05 | 1.04E-06 |
| Metabolism of serotonin | 10 | 1 | 99.074 | 14.286 | 2.49E-07 | 9.15E-05 | 1.04E-06 |
| Defective MAOA causes BRUNS | 4 | 1 | 99.074 | 14.286 | 2.49E-07 | 9.15E-05 | 1.04E-06 |
| Serotonin receptors | 10 | 1 | 99.074 | 14.286 | 2.49E-07 | 9.15E-05 | 1.04E-06 |
| Serotonin Neurotransmitter Release Cycle | 5 | 1 | 99.074 | 14.286 | 2.49E-07 | 9.15E-05 | 1.04E-06 |
| Heroin Action Pathway | 11 | 1 | 99.074 | 14.286 | 2.49E-07 | 9.15E-05 | 1.04E-06 |
| Citalopram Action Pathway | 22 | 1 | 99.074 | 14.286 | 2.49E-07 | 9.15E-05 | 1.04E-06 |
| Excitatory Neural Signalling Through 5-HTR 4 and Serotonin | 6 | 1 | 99.074 | 14.286 | 2.49E-07 | 9.15E-05 | 1.04E-06 |
| Excitatory Neural Signalling Through 5-HTR 6 and Serotonin | 6 | 1 | 99.074 | 14.286 | 2.49E-07 | 9.15E-05 | 1.04E-06 |
| Excitatory Neural Signalling Through 5-HTR 7 and Serotonin | 6 | 1 | 99.074 | 14.286 | 2.49E-07 | 9.15E-05 | 1.04E-06 |
| 3-Methylthiofentanyl Action Pathway | 9 | 1 | 99.074 | 14.286 | 2.49E-07 | 9.15E-05 | 1.04E-06 |
| Alfentanil Action Pathway | 9 | 1 | 99.074 | 14.286 | 2.49E-07 | 9.15E-05 | 1.04E-06 |
| Alvimopan Action Pathway | 9 | 1 | 99.074 | 14.286 | 2.49E-07 | 9.15E-05 | 1.04E-06 |
| Anileridine Action Pathway | 9 | 1 | 99.074 | 14.286 | 2.49E-07 | 9.15E-05 | 1.04E-06 |
| Benzocaine Action Pathway | 9 | 1 | 99.074 | 14.286 | 2.49E-07 | 9.15E-05 | 1.04E-06 |
| Bupivacaine Action Pathway | 9 | 1 | 99.074 | 14.286 | 2.49E-07 | 9.15E-05 | 1.04E-06 |
| Buprenorphine Action Pathway | 9 | 1 | 99.074 | 14.286 | 2.49E-07 | 9.15E-05 | 1.04E-06 |
| Carfentanil Action Pathway | 8 | 1 | 99.074 | 14.286 | 2.49E-07 | 9.15E-05 | 1.04E-06 |
| Chloroprocaine Action Pathway | 9 | 1 | 99.074 | 14.286 | 2.49E-07 | 9.15E-05 | 1.04E-06 |
| Cocaine Action Pathway | 9 | 1 | 99.074 | 14.286 | 2.49E-07 | 9.15E-05 | 1.04E-06 |
| Codeine Action Pathway | 17 | 1 | 99.074 | 14.286 | 2.49E-07 | 9.15E-05 | 1.04E-06 |
| Desipramine Action Pathway | 14 | 1 | 99.074 | 14.286 | 2.49E-07 | 9.15E-05 | 1.04E-06 |
| Dezocine Action Pathway | 9 | 1 | 99.074 | 14.286 | 2.49E-07 | 9.15E-05 | 1.04E-06 |
| Dibucaine Action Pathway | 9 | 1 | 99.074 | 14.286 | 2.49E-07 | 9.15E-05 | 1.04E-06 |
| Dihydromorphine Action Pathway | 9 | 1 | 99.074 | 14.286 | 2.49E-07 | 9.15E-05 | 1.04E-06 |
| Dimethylthiambutene Action Pathway | 9 | 1 | 99.074 | 14.286 | 2.49E-07 | 9.15E-05 | 1.04E-06 |
| Diphenoxylate Action Pathway | 9 | 1 | 99.074 | 14.286 | 2.49E-07 | 9.15E-05 | 1.04E-06 |
| Escitalopram Action Pathway | 9 | 1 | 99.074 | 14.286 | 2.49E-07 | 9.15E-05 | 1.04E-06 |
| Ethylmorphine Action Pathway | 9 | 1 | 99.074 | 14.286 | 2.49E-07 | 9.15E-05 | 1.04E-06 |
| Fentanyl Action Pathway | 9 | 1 | 99.074 | 14.286 | 2.49E-07 | 9.15E-05 | 1.04E-06 |
| Fluoxetine Action Pathway | 15 | 1 | 99.074 | 14.286 | 2.49E-07 | 9.15E-05 | 1.04E-06 |
| Hydrocodone Action Pathway | 9 | 1 | 99.074 | 14.286 | 2.49E-07 | 9.15E-05 | 1.04E-06 |
| Hydromorphone Action Pathway | 9 | 1 | 99.074 | 14.286 | 2.49E-07 | 9.15E-05 | 1.04E-06 |
| Imipramine Action Pathway | 16 | 1 | 99.074 | 14.286 | 2.49E-07 | 9.15E-05 | 1.04E-06 |
| Ketobemidone Action Pathway | 9 | 1 | 99.074 | 14.286 | 2.49E-07 | 9.15E-05 | 1.04E-06 |
| Levallorphan Action Pathway | 9 | 1 | 99.074 | 14.286 | 2.49E-07 | 9.15E-05 | 1.04E-06 |
| Levobupivacaine Action Pathway | 9 | 1 | 99.074 | 14.286 | 2.49E-07 | 9.15E-05 | 1.04E-06 |
| Levomethadyl Acetate Action Action Pathway | 9 | 1 | 99.074 | 14.286 | 2.49E-07 | 9.15E-05 | 1.04E-06 |
| Levorphanol Action Pathway | 9 | 1 | 99.074 | 14.286 | 2.49E-07 | 9.15E-05 | 1.04E-06 |
| Lidocaine (Local Anaesthetic) Action Pathway | 20 | 1 | 99.074 | 14.286 | 2.49E-07 | 9.15E-05 | 1.04E-06 |
| Mepivacaine Action Pathway | 9 | 1 | 99.074 | 14.286 | 2.49E-07 | 9.15E-05 | 1.04E-06 |
| Methadone Action Pathway | 14 | 1 | 99.074 | 14.286 | 2.49E-07 | 9.15E-05 | 1.04E-06 |
| Methadyl Acetate Action Pathway | 9 | 1 | 99.074 | 14.286 | 2.49E-07 | 9.15E-05 | 1.04E-06 |
| Morphine Action Pathway | 14 | 1 | 99.074 | 14.286 | 2.49E-07 | 9.15E-05 | 1.04E-06 |
| Nalbuphine Action Pathway | 9 | 1 | 99.074 | 14.286 | 2.49E-07 | 9.15E-05 | 1.04E-06 |
| Naloxone Action Pathway | 9 | 1 | 99.074 | 14.286 | 2.49E-07 | 9.15E-05 | 1.04E-06 |
| Naltrexone Action Pathway | 9 | 1 | 99.074 | 14.286 | 2.49E-07 | 9.15E-05 | 1.04E-06 |
| Nicotine Action Pathway | 28 | 1 | 99.074 | 14.286 | 2.49E-07 | 9.15E-05 | 1.04E-06 |
| Oxybuprocaine Action Pathway | 9 | 1 | 99.074 | 14.286 | 2.49E-07 | 9.15E-05 | 1.04E-06 |
| Oxycodone Action Pathway | 9 | 1 | 99.074 | 14.286 | 2.49E-07 | 9.15E-05 | 1.04E-06 |
| Oxymorphone Action Pathway | 9 | 1 | 99.074 | 14.286 | 2.49E-07 | 9.15E-05 | 1.04E-06 |
| Pentazocine Action Pathway | 9 | 1 | 99.074 | 14.286 | 2.49E-07 | 9.15E-05 | 1.04E-06 |
| Prilocaine Action Pathway | 9 | 1 | 99.074 | 14.286 | 2.49E-07 | 9.15E-05 | 1.04E-06 |
| Procaine Action Pathway | 9 | 1 | 99.074 | 14.286 | 2.49E-07 | 9.15E-05 | 1.04E-06 |
| Proparacaine Action Pathway | 9 | 1 | 99.074 | 14.286 | 2.49E-07 | 9.15E-05 | 1.04E-06 |
| Propoxyphene Action Pathway | 9 | 1 | 99.074 | 14.286 | 2.49E-07 | 9.15E-05 | 1.04E-06 |
| Remifentanil Action Pathway | 9 | 1 | 99.074 | 14.286 | 2.49E-07 | 9.15E-05 | 1.04E-06 |
| Ropivacaine Action Pathway | 9 | 1 | 99.074 | 14.286 | 2.49E-07 | 9.15E-05 | 1.04E-06 |
| Sufentanil Action Pathway | 9 | 1 | 99.074 | 14.286 | 2.49E-07 | 9.15E-05 | 1.04E-06 |
| Tramadol Action Action Pathway | 9 | 1 | 99.074 | 14.286 | 2.49E-07 | 9.15E-05 | 1.04E-06 |
| G alpha (i) signaling events | 91 | 1 | 99.074 | 14.286 | 2.49E-07 | 9.15E-05 | 1.04E-06 |
| G alpha (s) signaling events | 25 | 1 | 99.074 | 14.286 | 2.49E-07 | 9.15E-05 | 1.04E-06 |
| Melatonin metabolism and effects | 19 | 1 | 99.074 | 14.286 | 2.49E-07 | 9.15E-05 | 1.04E-06 |
| Serotonin receptor 4/6/7 and NR3C signaling | 2 | 1 | 99.074 | 14.286 | 2.49E-07 | 9.15E-05 | 1.04E-06 |
| Monoamine GPCRs | 7 | 1 | 99.074 | 14.286 | 2.49E-07 | 9.15E-05 | 1.04E-06 |
| Monoamine transport | 14 | 1 | 99.074 | 14.286 | 2.49E-07 | 9.15E-05 | 1.04E-06 |
| 7q11.23 copy number variation syndrome | 19 | 1 | 99.074 | 14.286 | 2.49E-07 | 9.15E-05 | 1.04E-06 |
| MECP2 and associated Rett syndrome | 11 | 1 | 99.074 | 14.286 | 2.49E-07 | 9.15E-05 | 1.04E-06 |
| GPCRs, class A rhodopsin-like | 9 | 1 | 99.074 | 14.286 | 2.49E-07 | 9.15E-05 | 1.04E-06 |
| Tryptophan catabolism leading to NAD+ production | 23 | 1 | 99.074 | 14.286 | 2.49E-07 | 9.15E-05 | 1.04E-06 |
| Response to elevated platelet cytosolic Ca ions | 11 | 1 | 99.074 | 14.286 | 2.49E-07 | 9.15E-05 | 1.04E-06 |
| Serotonin HTR1 group and FOS pathway | 6 | 1 | 99.074 | 14.286 | 2.49E-07 | 9.15E-05 | 1.04E-06 |
| Serotonin transporter activity | 3 | 1 | 99.074 | 14.286 | 2.49E-07 | 9.15E-05 | 1.04E-06 |
| Serotonin receptor 2 and ELK-SRF/GATA4 signaling | 3 | 1 | 99.074 | 14.286 | 2.49E-07 | 9.15E-05 | 1.04E-06 |
| Serotonin and anxiety-related events | 2 | 1 | 99.074 | 14.286 | 2.49E-07 | 9.15E-05 | 1.04E-06 |
| Metabolism | 1369 | 15 | 51.273 | 14.286 | 1.69E-05 | 0.0047218 | 6.97E-05 |
| Tryptophan metabolism | 53 | 3 | 85.156 | 14.286 | 2.06E-05 | 0.0057566 | 8.44E-05 |
| Amino acid metabolism | 109 | 6 | 68.486 | 14.286 | 2.63E-05 | 0.0073167 | 0.00010643 |
| Biochemical pathways: part I | 445 | 13 | 53.75 | 14.286 | 2.87E-05 | 0.0079426 | 0.00011469 |
| Biogenic amine synthesis | 17 | 4 | 71.925 | 14.286 | 4.29E-05 | 0.011831 | 0.00016606 |
| Sudden infant death syndrome (SIDS) susceptibility pathways | 14 | 4 | 71.925 | 14.286 | 4.29E-05 | 0.011831 | 0.00016606 |
| Metabolism of amine-derived hormones | 36 | 4 | 71.925 | 14.286 | 4.29E-05 | 0.011831 | 0.00016606 |
| Metabolism of amino acids and derivatives | 283 | 8 | 62.885 | 14.286 | 7.80E-05 | 0.021283 | 0.00029885 |
| Neurotransmitter disorders | 23 | 5 | 66.968 | 14.286 | 0.00016385 | 0.044566 | 0.00061526 |
| Biosynthesis and regeneration of tetrahydrobiopterin and catabolism of phenylalanine | 31 | 5 | 66.968 | 14.286 | 0.00016385 | 0.044566 | 0.00061526 |
| Neurotransmitter clearance | 32 | 2 | 72.787 | 14.286 | 0.00053961 | 0.1457 | 0.0020058 |
| Disease | 470 | 8 | 46.947 | 14.286 | 0.00087626 | 0.23571 | 0.0032246 |
| Neurotransmitter release cycle | 46 | 2 | 65.307 | 14.286 | 0.0012796 | 0.34294 | 0.0046167 |
| Synaptic vesicle pathway | 7 | 2 | 65.307 | 14.286 | 0.0012796 | 0.34294 | 0.0046167 |
| Signal Transduction | 345 | 2 | 65.053 | 14.286 | 0.0014043 | 0.37355 | 0.0046468 |
| Signaling by GPCR | 236 | 2 | 65.053 | 14.286 | 0.0014043 | 0.37355 | 0.0046468 |
| Class A/1 (Rhodopsin-like receptors) | 154 | 2 | 65.053 | 14.286 | 0.0014043 | 0.37355 | 0.0046468 |
| GPCR downstream signalling | 156 | 2 | 65.053 | 14.286 | 0.0014043 | 0.37355 | 0.0046468 |
| GPCR ligand binding | 211 | 2 | 65.053 | 14.286 | 0.0014043 | 0.37355 | 0.0046468 |
| G alpha (q) signalling events | 63 | 2 | 65.053 | 14.286 | 0.0014043 | 0.37355 | 0.0046468 |
| Amine ligand-binding receptors | 65 | 2 | 65.053 | 14.286 | 0.0014043 | 0.37355 | 0.0046468 |
| G alpha (q) signaling events | 53 | 2 | 65.053 | 14.286 | 0.0014043 | 0.37355 | 0.0046468 |
| Transport of vitamins, nucleosides and related molecules | 62 | 2 | 66.125 | 14.286 | 0.0014143 | 0.37355 | 0.0046468 |
| Transport of nucleosides and free purine and pyrimidine bases across the plasma membrane | 21 | 2 | 66.125 | 14.286 | 0.0014143 | 0.37355 | 0.0046468 |
| Biological oxidations | 317 | 3 | 55.847 | 14.286 | 0.0015986 | 0.40924 | 0.0052061 |
| Organic cation/anion/zwitterion transport | 34 | 2 | 67.356 | 14.286 | 0.0019409 | 0.49492 | 0.0062652 |
| Transport of small molecules | 208 | 6 | 49.229 | 14.286 | 0.0021602 | 0.54869 | 0.0068531 |
| SLC-mediated transmembrane transport | 155 | 6 | 49.229 | 14.286 | 0.0021602 | 0.54869 | 0.0068531 |
| Transport of bile salts and organic acids, metal ions and amine compounds | 76 | 4 | 55.833 | 14.286 | 0.0024794 | 0.62481 | 0.0077985 |
| Na+/Cl- dependent neurotransmitter transporters | 32 | 3 | 62.584 | 14.286 | 0.0025996 | 0.65249 | 0.0081071 |
| Sphingolipid de novo biosynthesis | 36 | 2 | 47.85 | 14.286 | 0.0038502 | 0.96256 | 0.011907 |
| SLC transporter disorders | 80 | 4 | 40.531 | 14.286 | 0.0060775 | 1 | 0.018484 |
| Disorders of transmembrane transporters | 98 | 4 | 40.531 | 14.286 | 0.0060775 | 1 | 0.018484 |
| Tryptophan catabolism | 33 | 2 | 57.067 | 14.286 | 0.0087564 | 1 | 0.026248 |
| Transmission across Chemical Synapses | 77 | 3 | 58.945 | 14.286 | 0.0088444 | 1 | 0.026248 |
| Neuronal System | 77 | 3 | 58.945 | 14.286 | 0.0088444 | 1 | 0.026248 |
| Metabolism of proteins | 295 | 3 | 42.35 | 14.286 | 0.011512 | 1 | 0.033357 |
| Post-translational protein modification | 169 | 3 | 42.35 | 14.286 | 0.011512 | 1 | 0.033357 |
| Transport of inorganic cations/anions and amino acids/oligopeptides | 52 | 3 | 42.35 | 14.286 | 0.011512 | 1 | 0.033357 |
| Glyoxylate metabolism and glycine degradation | 38 | 1 | 66.001 | 14.286 | 0.014268 | 1 | 0.040704 |
| Glyoxylate metabolism | 15 | 1 | 66.001 | 14.286 | 0.014268 | 1 | 0.040704 |
| GABA metabolism (aka GHB) | 27 | 3 | 41.188 | 14.286 | 0.016373 | 1 | 0.046348 |
| Citric Acid Cycle | 26 | 1 | 63.007 | 14.286 | 0.018676 | 1 | 0.049446 |
| 2-ketoglutarate dehydrogenase complex deficiency | 26 | 1 | 63.007 | 14.286 | 0.018676 | 1 | 0.049446 |
| Congenital lactic acidosis | 26 | 1 | 63.007 | 14.286 | 0.018676 | 1 | 0.049446 |
| Fumarase deficiency | 26 | 1 | 63.007 | 14.286 | 0.018676 | 1 | 0.049446 |
| Mitochondrial complex II deficiency | 26 | 1 | 63.007 | 14.286 | 0.018676 | 1 | 0.049446 |
| Pyruvate dehydrogenase deficiency (E2) | 26 | 1 | 63.007 | 14.286 | 0.018676 | 1 | 0.049446 |
| Pyruvate dehydrogenase deficiency (E3) | 26 | 1 | 63.007 | 14.286 | 0.018676 | 1 | 0.049446 |
| Amino acid metabolism pathway excerpt: histidine catabolism extension | 17 | 1 | 63.007 | 14.286 | 0.018676 | 1 | 0.049446 |
| TCA cycle (aka Krebs or citric acid cycle) | 23 | 1 | 63.007 | 14.286 | 0.018676 | 1 | 0.049446 |

Table S5: Univariate ROC analysis of metabolites (AUC < 1, Maximum FPR: 0.2)

| **Metabolites** | **AUC** | **Pval** | **FC** | **clusters** |
| --- | --- | --- | --- | --- |
| (2E)-Decenoyl-ACP | 1 | 0.0155 | 1.08 | 5 |
| 3-Hydroxyphenylacetic acid | 1 | 0.0072 | -1.27 | 4 |
| Catechol | 1 | 0.0195 | 0.87 | 5 |
| D-2-Hydroxyglutaric acid | 1 | 0.0172 | -0.86 | 4 |
| Glycolic acid | 1 | 0.0005 | 1.54 | 5 |
| Hippuric acid | 1 | 0.0003 | -1.17 | 4 |
| Homovanillic acid | 1 | 0.0045 | 0.66 | 5 |
| Indole-3-acetaldehyde | 1 | 0.0067 | 2.76 | 5 |
| Indolelactate | 1 | 0.0254 | -1.10 | 4 |
| N-Acetylneuraminic acid | 1 | 0.0643 | 1.30 | 3 |
| Phytosphingosine | 1 | 0.0020 | 0.75 | 5 |
| Shikimic acid | 1 | 0.0027 | 1.99 | 5 |

Note: Due to the pilot nature of this study (N=2), ROC results are purely descriptive and intended for hypothesis generation. AUC values of 1.0 reflect the high contrast in this specific dataset and are subject to overfitting; they do not imply validated diagnostic performance.

## Table S6: Key metabolites from multivariate ROC analysis

| **Metabolites** | **Rank Freq.** | **Importance** | **As_after** | **RDA_after** |
| --- | --- | --- | --- | --- |
| Shikimic acid | 0.5 | 0.009 | High | Low |
| D-2-Hydroxyglutaric acid | 0.38 | 0.008 | Low | High |
| Hippuric acid | 0.34 | 0.008 | Low | High |
| Catechol | 0.32 | 0.008 | High | Low |
| Norvaline | 0.24 | 0.006 | High | Low |
| Phytosphingosine | 0.1 | 0.006 | High | Low |
| Indole-3-acetaldehyde | 0.06 | 0.005 | High | Low |
| 3-Hydroxyphenylacetic acid | 0.06 | 0.005 | Low | High |

Note: Due to the pilot nature of this study (N=2), ROC results are purely descriptive and intended for hypothesis generation. AUC values of 1.0 reflect the high contrast in this specific dataset and are subject to overfitting; they do not imply validated diagnostic performance.

# **Supplementary figures**


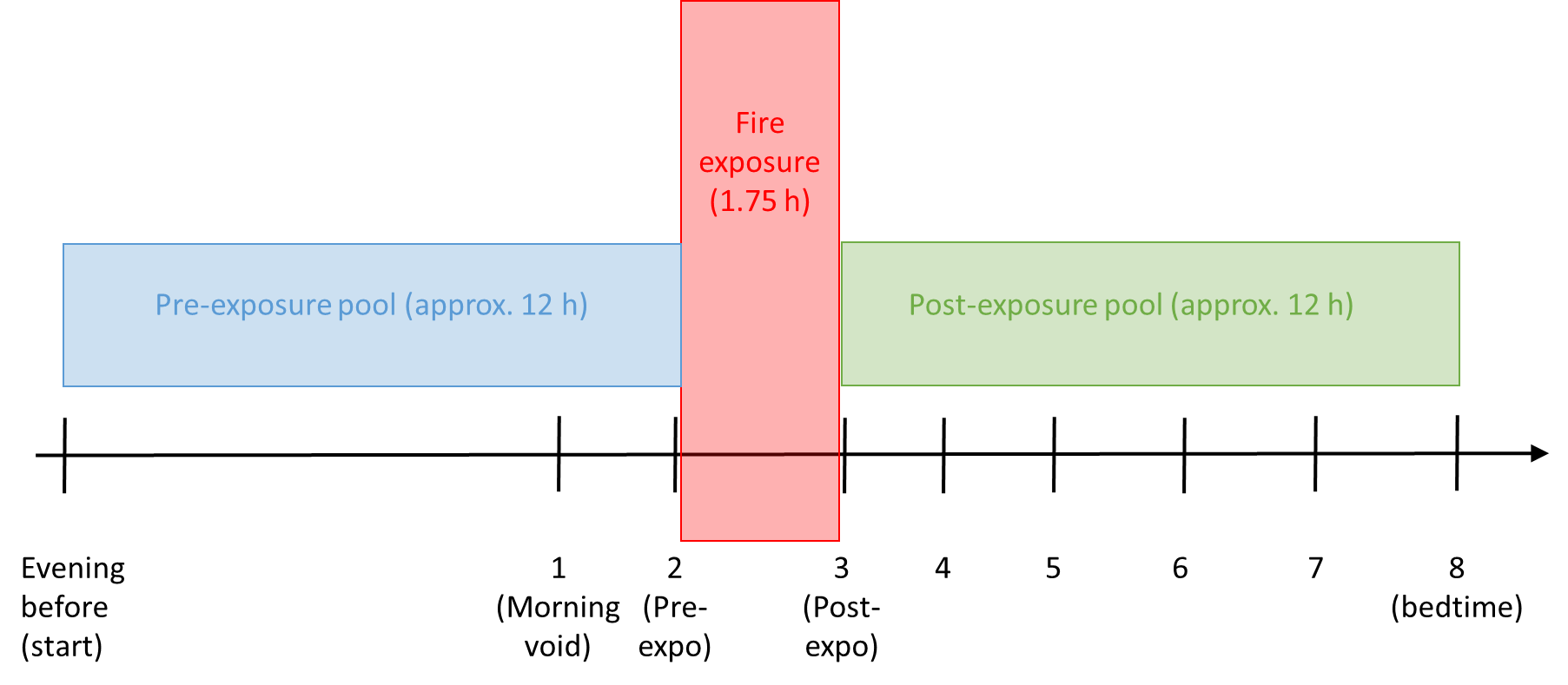


## Figure S1: Schematic representation of the study design and 24-hour urine sampling protocol.

The sampling period covered approximately 24 hours, starting the evening prior to the exercise. Participants collected every individual urine void at nine predefined time points, beginning **after** the last urination before going to bed. Bladder voidings apart from the predefined time points were pooled with the next scheduled urine sample. These timed samples were subsequently combined into two composite samples per session—a pre-exposure pool and a post-exposure pool—using volume-weighted aliquots to ensure a representative metabolic signal. The firefighting training (RDA) lasted approximately 1.75 hours. The physical exertion scenario (AS) followed an identical temporal sampling scheme to serve as an active control for circadian and metabolic baseline variations. (Adapted from (Rossbach et al. 2020)).


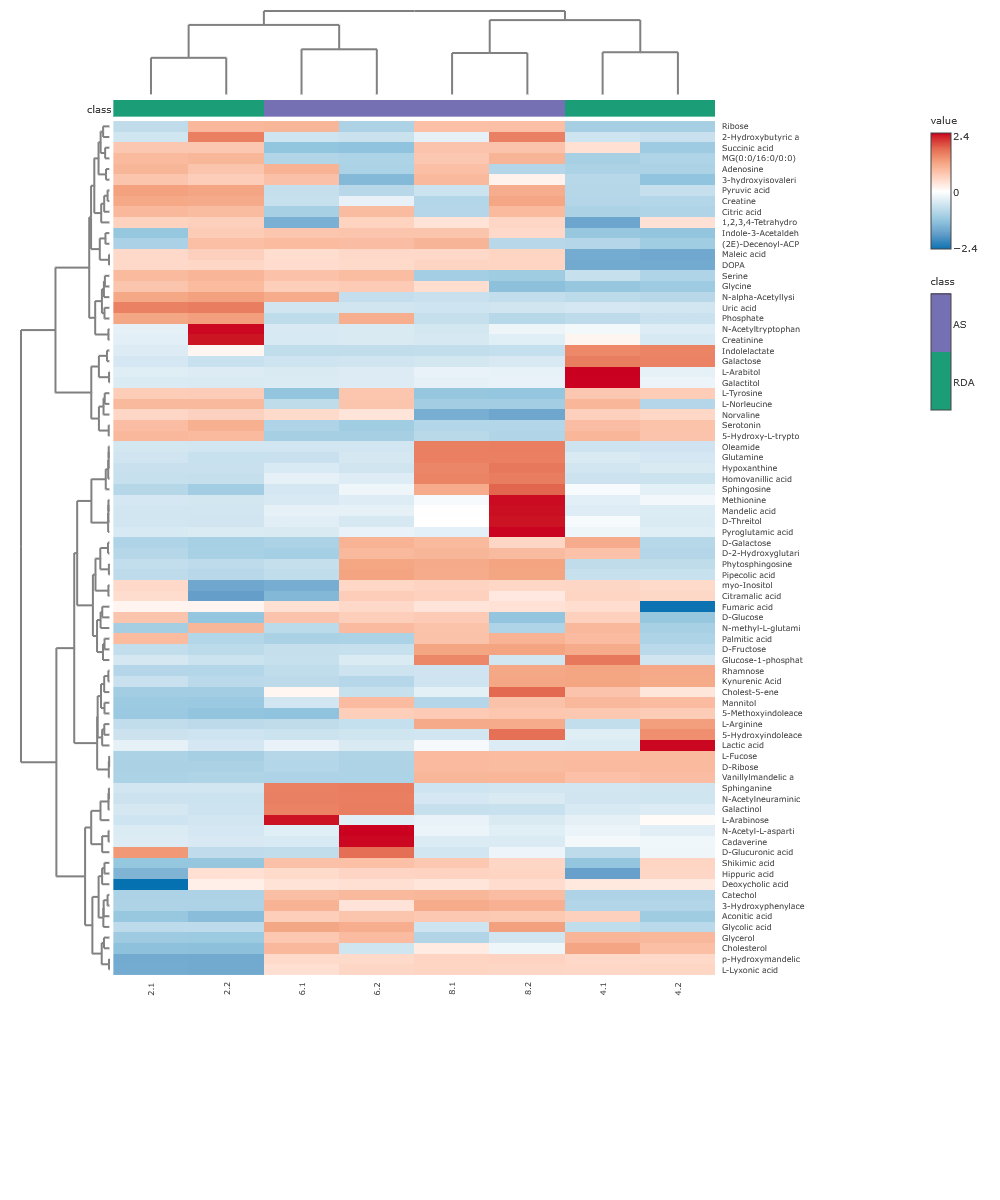


Figure S2: Heatmap of metabolite log2(FC) values. The analysis shows hierarchical clustering of samples (columns) and metabolites (rows).


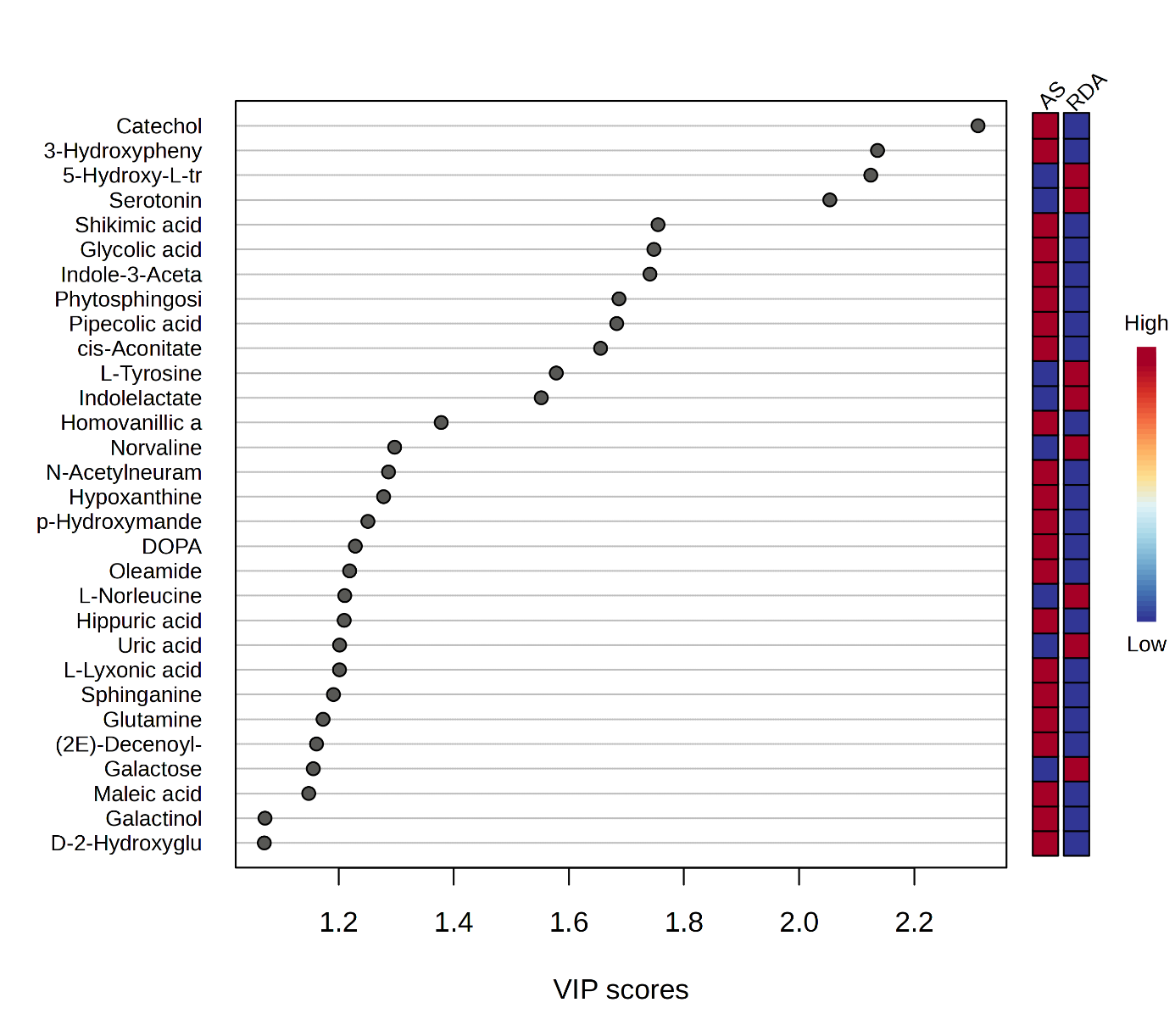


Figure S3: PLS-DA VIP scores plot. The plot shows the top metabolites ranked by their contribution to the separation between the fire exposure (RDA) and active control (AS) groups. A VIP score greater than 1 signifies metabolites with significant importance to the PLS-DA model.


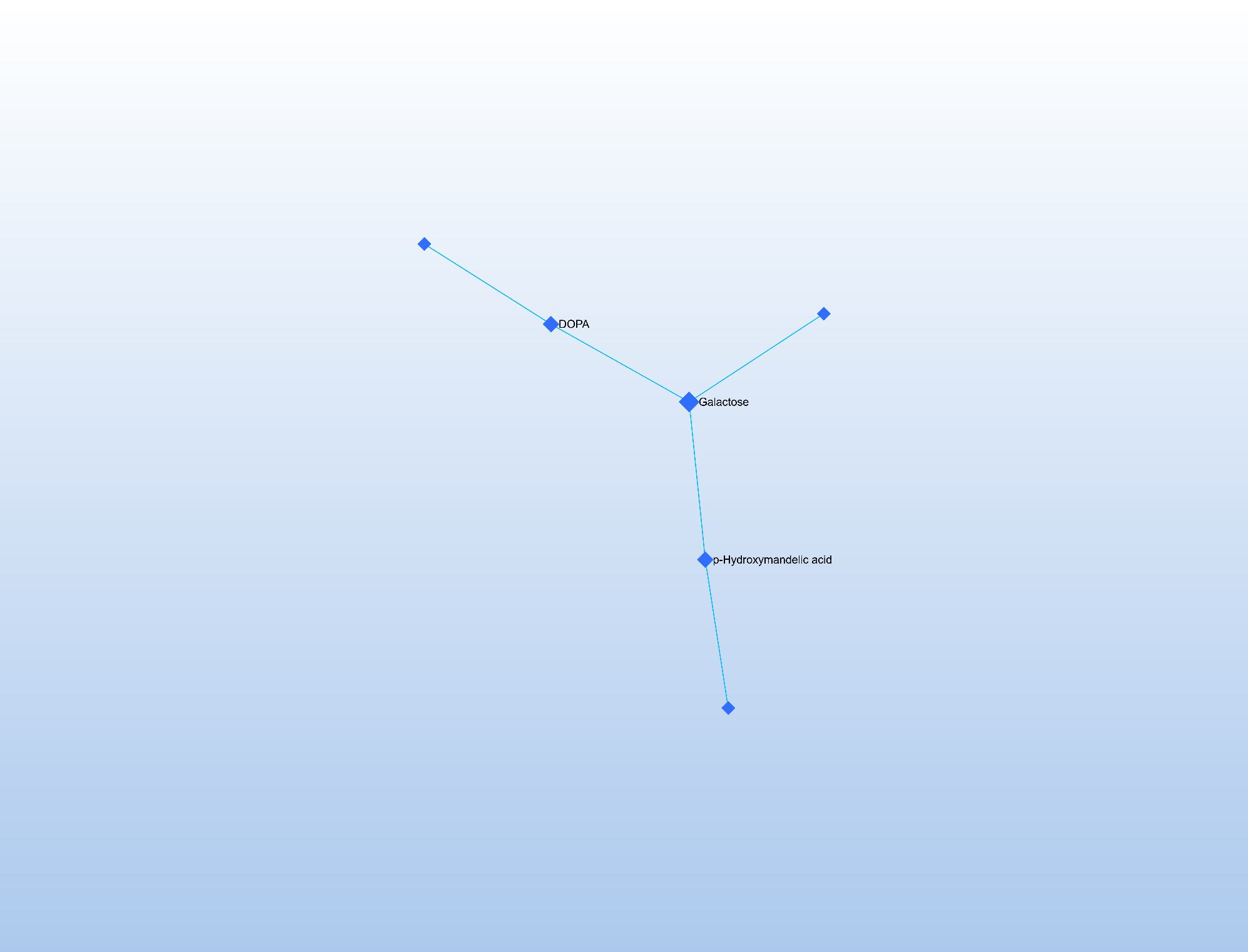


Figure S4: Metabolite correlation network for the RDA group. Nodes represent metabolites, and edges show correlations. The thickness of an edge corresponds to the correlation strength. Blue edges indicate a positive correlation between metabolites.

Rossbach B, Wollschlager D, Letzel S, Gottschalk W, Muttray A (2020) Internal exposure of firefighting instructors to polycyclic aromatic hydrocarbons (PAH) during live fire training. Toxicol Lett 331:102–111 doi:10.1016/j.toxlet.2020.05.024
